# Silent and Cold: A Case of Bradycardia Associated With Isolated Hypothermia

**DOI:** 10.7759/cureus.60991

**Published:** 2024-05-24

**Authors:** Noah Ene, Elizabeth Colvin, Manoj Bhandari

**Affiliations:** 1 Internal Medicine, Cape Fear Valley Health, Fayetteville, USA; 2 Internal Medicine, Campbell University School of Osteopathic Medicine, Fayetteville, USA; 3 Cardiology, Cape Fear Valley Medical Center, Fayetteville, USA

**Keywords:** hypotension, beta blocker, ecg, altered mental status, cardiac arrhythmia, osborn waves, hypothermia, bradycardia

## Abstract

Sinus bradycardia is defined as a heart rate of less than 60 beats per minute and can occur as an adaptive response but can also be pathologic. Sinus bradycardia can be a normal finding in children, individuals who exercise often, and as a physiologic response during sleep. Pathologic causes of sinus bradycardia include sinus node dysfunction, medications, acute myocardial infarction, heart failure, obstructive sleep apnea, exaggerated vagal activity, increased intracranial hypertension, infection, hypothyroidism, hypothermia, anorexia nervosa, and prolonged hypoxia. When pathologic, addressing the underlying cause will lead to an improvement in heart rate. Here, we present a case of sinus bradycardia in a 61-year-old female with hypothermia. Evaluation for common causes of bradycardia including cardiac evaluation was unremarkable. Treatment of hypothermia led to the resolution of bradycardia. The importance of the case is to help clinicians recognize hypothermia as a cause of bradycardia.

## Introduction

Sinus bradycardia is defined as a heart rate less than 60 beats per minute and can occur as an adaptive response but can also be pathologic [1]. During sleep, sinus bradycardia, especially in children, is seen as a normal finding, even with a heart rate as low as 30 beats per minute [1]. Medications are a common cause of bradycardia amongst which calcium channel blockers and beta blockers are usual culprits [2-4]. Pathologic causes of sinus bradycardia include sinus node dysfunction, medications, acute myocardial infarction, heart failure, obstructive sleep apnea, exaggerated vagal activity, increased intracranial hypertension, infection, hypothyroidism, hypothermia, anorexia nervosa, and prolonged hypoxia [2,5]. Hypothermia can be classified into three stages namely: mild (32-35 °C), moderate (28-32 °C), and severe hypothermia (<28 °C). A significant ECG finding associated with hypothermia is Osborn (J) waves which have been reported to occur in 36% of hypothermic patients and are usually seen when the temperature falls below 30 °C [6,7]. We present a case of a 61-year-old female with prolonged bradycardia who was later found to be hypothermic given the presence of Osborn (J) waves on ECG.

## Case presentation

A 61-year-old female with a history of mental disability, congestive heart failure, essential hypertension, depression, systemic lupus erythematosus (SLE) with class IV nephritis presented to an emergency room from a group home with decreased mental status. Home medications were hydroxychloroquine, metoprolol tartrate, omeprazole, clonazepam, risperidone, isosorbide dinitrate, benztropine, citalopram, bisacodyl, and trazodone. Vital signs were as follows: oral temperature 36 °C, respiratory rate 16 breaths/min, HR 54 bpm, blood pressure 164/97 mmHg, and oxygen saturation 97% at ambient air. Physical examination revealed a comatose patient, hypoventilation, and cold extremities. There was bradycardia, reduced air entry in lung bases, and normal heart sounds. Laboratory results are summarized in Tables 1-5 below.

**Table 1:** Complete Blood Count

Complete blood count	Reference ranges	Patient’s lab values
White blood cell count	4.5 - 12.5 x10^3^/uL	5.6 x10^3^/uL
Red blood cell count	4.70 - 6.10 x10^6^/uL	3.9 X10^6^/uL
Hemoglobin	13.5 - 18.0 g/dL	11.5 g/dL
Hematocrit	40.5 - 54.0 %	36.5 %
Mean corpuscular volume	80.0 - 95.0 fL	93.6 fL
Mean corpuscular hemoglobin concentration	31.0-36.0 g/dL	31.5 g/dL
Platelets	150 - 450 x10^3^/uL	113 x10^3^/uL

**Table 2:** Complete Metabolic Panel

Comprehensive metabolic panel	Reference ranges	Patient's lab values
Sodium	136 - 145 mmol/L	137 mmol/L
Potassium	3.4 - 4.9 mmol/L	3.5 mmol/L
Chloride	98 - 107 mmol/L	96 mmol/L
Carbon dioxide	21 - 32 mmol/L	31 mmol/L
Anion gap	1 - 11 mmol/L	10 mmol/L
Blood urea nitrogen	7 - 25 mg/dL	21 mg/dL
Creatinine	0.60 - 1.30 mg/dL	0.61 mg/dL
Estimated glomerular filtration rate	>60.0 mL/min/1.73m^2^	> 60 mL/min/1.73m^2^
Glucose	74 - 109 mg/dL	73 mg/dL
Calcium	8.6 - 10.2 mg/dL	9.5 mg/dL
Alkaline phosphatase	30 - 105 U/L	83 U/L
Albumin	3.5 - 5.7 g/dL	3.1 g/dL
Total protein	6.4 - 8.9 g/dL	7.1 g/ dL
Aspartate aminotransferase	13 -39 U/L	26 U/L
Alanine transaminase	7-52 U/L	20 U/L
Bilirubin total	0.3 - 1.0 mg/dL	0.8 mg/dL
Lactate	0.8 mg/dL	0.5 - 2.0 mg/dL
Phosphorus	2.4 - 4.7 mg/dL	1.3 mg/dL
Magnesium	1.9 -2.7 mg/dL	1.2 mg/dL

**Table 3:** Thyroid Studies

Thyroid Studies	Reference ranges	Patient’s lab values
Thyroid stimulating Hormone	0.450-5.330 ulU/mL	8.176 ulU/mL
Free T4	0.61 -1.12ng/dL	1.12 ng/dL

**Table 4:** Urinalysis Ur: urine

Urinalysis	Reference ranges	Patient’s lab values
Color, Ur	Amber	Light Yellow
Clarity, Ur	Clear	Clear
Specific gravity, Ur	1.010 - 1.025	1.010
pH Ur	5.0 -8.0	7.5
WBC esterase	Negative	Negative
Nitrite, Ur	Negative	Negative
Protein, Ur	Negative	Negative
Glucose, Ur	Negative	Negative
Ketones, Ur	Negative	1+
Urobilinogen Ur	Normal	Normal
Bilirubin, Ur	Negative	Negative
Blood, Ur	Negative	Negative
RBC, U	0-3 /HPF	3 /HPF
WBC, Ur	0-10 / HPF	3 /HPF
Squamous epithelial, Ur	<15 -20 /HPF	10 /HPF
Bacteria, Ur	None seen /HPF	1+
Trans epithelial, Ur	0 /HPF	<1 HPF
Mucus, Ur	None seen /LPF	Occasional
Granular casts, Ur	<1 HPF	None
Hyaline casts, Ur	<1 LPF	None

**Table 5:** Urine toxicology Ur: urine; Qual: qualititative; THC: Tetrahydrocannabinol; PCP: phencyclidine

TOX, Urine	Reference ranges	Patient’s lab values
Amphetamine screen, Ur	Negative	Negative
Barbiturate screen, Ur	Negative	Negative
Benzodiazepine, Ur Qual	Negative	Negative
Opiate, Ur	Negative	Negative
Cocaine screen, Ur	Negative	Negative
THC screen, Ur	Negative	Negative
PCP screen, Ur	Negative	Negative
Methadone	Negative	Negative

As initial management, the patient was intubated and admitted to the ICU, electrolytes were corrected, and metoprolol tartrate was held.

The patient continued to be bradycardic with a heart rate in the low 30s. Sedation was decreased, and a dose of atropine 1 mg was given. The patient’s heart rate did not improve, and transvenous cardiac pacing (TVP) was placed with the rate set at 80 bpm. The patient then became hypotensive, and an epinephrine drip was started. Blood cultures came back negative.

**Figure 1:**
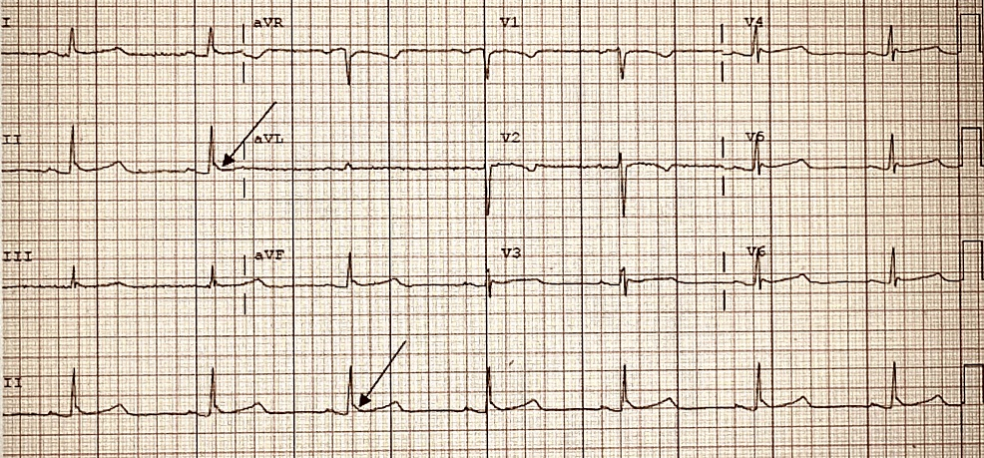
ECG tracing with a HR of 42 showing sinus bradycardia, borderline PR interval (227), and Osborn waves (black arrows) suggesting hypothermia

ICU consulted cardiology for evaluation of bradycardia. Cardiology ordered an ECG and an echocardiogram as part of an initial assessment. ECG demonstrated sinus bradycardia, HR 42, prolonged PR, QT interval, and Osborn waves, which were suggestive of hypothermia, as shown in Figure 1 below. Chest X-ray was negative for any acute abnormality. CT head without contrast was negative for bleeding or other acute intracranial processes. TTE indicated EF >55%, normal left ventricular systolic function, and no wall motion abnormality. A repeat temperature showed the patient was hypothermic with a temperature of 30.3 °C. As such, cardiology recommended hypothermia correction as management of bradycardia. The patient’s heart rate improved and was sustained in the 80s with normalization in body temperature. TVP was stopped.

## Discussion

Bradycardia can be a normal adaptive response or pathologic. When approaching a patient with a presumed pathologic presentation of bradycardia, assessing reversible causes is an important first step [1]. Certain medications including parasympathomimetic agents, sympatholytic drugs, sedatives, opioids, calcium channel blockers, lithium, ivabradine, cimetidine, digitalis, antiarrhythmic agents, chemotherapeutic agents, and organophosphate compounds can be potential causes of bradycardia [1]. In this presentation, the patient was receiving propofol, fentanyl, and Lopressor 50 mg twice daily. The combination of sedatives and beta-blockers could have exacerbated her bradycardia with underlying hypothermia.

Beta-blocker toxicity was an initial differential diagnosis for the cause of bradycardia in this patient. Hypotension and bradycardia are prominent with PR prolongation or any bradyarrhythmia seen on ECG [2,3]. The patient may also have hyperkalemia and hypoglycemia, but this diagnosis is mainly based on history and clinical findings [2,4]. Patients who ingest large amounts of beta-blockers that specifically have membrane stabilizing activity (MSA) have a higher risk of cardiovascular adverse effects. Carvedilol, betaxolol, and propranolol have MSA while Lopressor has low MSA [2]. In the presented case, there was no known instance of significant ingestion of Lopressor; however, this patient was mentally disabled, and an accurate history was difficult to attain. The patient remained hypotensive but there was no hyperkalemia or hypoglycemia to suggest beta-blocker toxicity. It is unlikely that the patient ingested a large amount of Lopressor because the patient lives in a group home and is not able to access her medication.

Sinus node dysfunction describes the inability of the sinoatrial (SA) node to generate a heart rate adequate enough to perfuse and provide for the physiologic needs of the human body [5]. Diagnosis is largely based on ECG findings which can be numerous including sinus bradycardia, sinus pauses, sinus arrest, prolonged RR, PR, QRS, and QT intervals, and supraventricular tachycardias. The underlying etiology of sinus node dysfunction is mostly attributed to SA node fibrosis, medications, and congenital heart diseases [5,6]. Fibrosis is the most common cause of dysfunction but medications like beta blockers, non-dihydropyridine calcium channel blockers, digoxin, antiarrhythmics, and acetylcholinesterase inhibitors can also cause SA node dysfunction. Familiar sinus node dysfunction is a rare cause of SA dysfunction typically seen in childhood. Other causes include trauma, infection, inflammation, and hypothermia. Our patient had no previous history of bradycardia, abnormal ECGs, or structural heart disease indicating an intrinsic cause of SA node dysfunction, however, her use of a beta-blocker and her hypothermic state were the most likely extrinsic causes.

Upon further investigation, the patient was found to be hypothermic. Hypothermia is defined as a core temperature below 35 °C and is divided into three different stages. Mild hypothermia includes a core temperature of 32-35 °C, moderate is 28-32 °C, and severe is <28 °C. The stage of hypothermia has a large impact on the clinical presentation and treatment [6,7]. Initial temperature taken on presentation showed an oral temperature of 36 °C, which is not considered hypothermic, however, repeat vitals did not include a temperature until after transfer and hours after the patient had arrived at the accepting hospital. The repeat temperature was 30.3 °C, indicating moderate hypothermia. A patient presenting with moderate hypothermia will portray lethargy, altered mental status, progressive bradycardia that is unresponsive to atropine, Osborn (J) waves on ECG, decreased oxygen consumption, and a lack of shivering [6,7]. 

The presented case experienced altered mental status which was attributed to her hypercapnic state. Shivering was not seen on examination and Osborn (J) waves were found on repeat ECG (Figure 1) later during her presentation. Osborn waves are commonly seen in hypothermia. Osborn waves have also been associated with early repolarization, hypercalcemia, Brugada syndrome, intracranial hemorrhage, and brain injury [6,7]. This case was uniquely difficult due to the inability to obtain an accurate history and, therefore, we relied on monitoring of vitals and a thorough physical examination. The initial temperature taken orally may not have accurately reflected the patient’s core temperature. In patients with suspected hypothermia, a rectal temperature is reasonable in a conscious patient and an esophageal temperature is the most accurate predictor in intubated patients [6]. The route of temperature was not documented for proceeding values but was serially monitored as the patient was warmed. Her heart rate positively correlated with an increase in temperature. The patient’s sinus bradycardia resolved once the patient was within normal physiologic temperature range. 

## Conclusions

Pathologic sinus bradycardia can be caused by structural heart abnormalities, medications, temperature changes, and heart failure, amongst others. When approaching a patient with sinus bradycardia of unknown etiology, assessing reversible causes is paramount. In a patient who is unable to give an accurate history, monitoring vital signs and conducting a thorough physical exam are crucial to assessing these reversible causes. If a patient is found to be hypothermic, a reversible cause of sinus bradycardia, brisk warming and continual monitoring for aberrant cardiac arrhythmias are important to reduce mortality. The case is unique given that the presence of Osborn waves on ECG led to the identification of hypothermia as a cause of the patient's bradycardia. More so, the patient was not hypothermic on presentation although they became hypothermic during hospitalization.
